# *catena*-Poly[[(*N*,*N*-diethyl­dithio­carbamato-κ^2^ *S*:*S*′)phenyl­bismuth(III)]-μ-chlorido]

**DOI:** 10.1107/S1600536808032820

**Published:** 2008-10-22

**Authors:** Liansheng Cui, Handong Yin, Minglei Yang, Li Quan, Daqi Wang

**Affiliations:** a College of Chemistry and Chemical Engineering, Liaocheng University, Shandong 252059, People’s Republic of China

## Abstract

In the title compound, [Bi(C_6_H_5_)(C_5_H_10_NS_2_)Cl]_*n*_, the Bi atom is coordinated by two S atoms of the dithio­carbamate ligand, one C atom of the phenyl group and one Cl atom in a four-coordinated tetra­hedral configuration. Mol­ecules are linked by Cl atoms to form a zigzag chain extending in the *c* direction.

## Related literature

For related literature see: Yin *et al.* (2003 ▶); Bardaji *et al.* (1994 ▶); Xu *et al.* (2001 ▶).
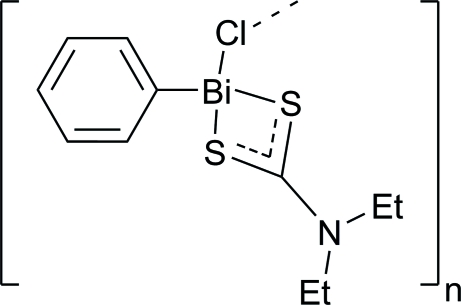


## Experimental

### 

#### Crystal data

[Bi(C_6_H_5_)(C_5_H_10_NS_2_)Cl]
*M* *_r_* = 469.79
Monoclinic, 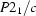

*a* = 9.2029 (9) Å
*b* = 18.1432 (17) Å
*c* = 9.0779 (8) Å
β = 106.811 (2)°
*V* = 1451.0 (2) Å^3^
*Z* = 4
Mo *K*α radiation
μ = 12.60 mm^−1^
*T* = 298 (2) K
0.23 × 0.22 × 0.21 mm

#### Data collection

Bruker SMART CCD area-detector diffractometer
Absorption correction: multi-scan (*SADABS*; Sheldrick, 2003 ▶) *T*_min_ = 0.160, *T*_max_ = 0.177 (expected range = 0.064–0.071)
6443 measured reflections
2444 independent reflections
1877 reflections with *I* > 2σ(*I*)
*R*_int_ = 0.095

#### Refinement

*R*[*F* ^2^ > 2σ(*F* ^2^)] = 0.056
*wR*(*F* ^2^) = 0.149
*S* = 0.92
2444 reflections
147 parameters
H-atom parameters constrained
Δρ_max_ = 2.04 e Å^−3^
Δρ_min_ = −2.84 e Å^−3^

### 

Data collection: *SMART* (Bruker, 2001 ▶); cell refinement: *SAINT* (Bruker, 2001 ▶); data reduction: *SAINT*; program(s) used to solve structure: *SHELXS97* (Sheldrick, 2008 ▶); program(s) used to refine structure: *SHELXL97* (Sheldrick, 2008 ▶); molecular graphics: *SHELXTL* (Sheldrick, 2008 ▶); software used to prepare material for publication: *SHELXTL*.

## Supplementary Material

Crystal structure: contains datablocks I, global. DOI: 10.1107/S1600536808032820/su2063sup1.cif

Structure factors: contains datablocks I. DOI: 10.1107/S1600536808032820/su2063Isup2.hkl

Additional supplementary materials:  crystallographic information; 3D view; checkCIF report

## Figures and Tables

**Fig. 1.**
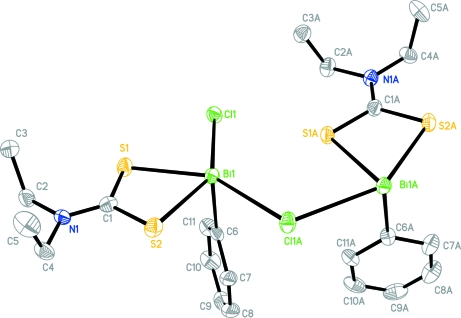
The moleculare structure of the title compound, showing the atom-numbering scheme and displacement ellipsoids drawn at the 50% probability level.

**Fig. 2.**
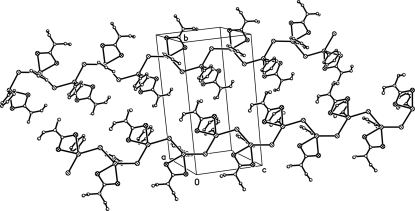
The crystal packing of the title compound, viewed approximately along the a axis.

## supplementary crystallographic information

### Comment

Dithiocarbamates have been known as effective ligands for many years. They can form chelates (Xu *et al.*, 2001) or act as bridging ligands (Bardaji *et al.*, 1994). However, the chemistry of main-group metal complexes with dithiocarbamates has been less extensively studied, and only a few reports describing bismuth dithiocarbamate complexes have appeared (Yin *et al.*, 2003). As a continuation of our interest in sulfur-containing ligands, we report here the synthesis and structure of the title compound.

The molecular structure of the title compound is illustrated in Fig. 1. The geometry of the Bi atom is four-coordinated tetrahedral. Two Bi—S bonds distance are not similar, the Bi1—S1 bond length is 2.647 (3) Å and the Bi1—S2 bond length is 2.690 (4) Å. The molecules are linked by the chlorine atom Cl1 [Bi1-Cl1 = 2.908 (3) Å and Bi1···Cl1^i^ = 2.920 (3); (i) = x, -y+0.5, z+0.5] to form a one-dimensional zig-zag polymer chain extending in the c direction (Fig. 2).

### Experimental

A mixture of (*N*,*N*-diethylcarbamato-1*k*^2^*S*:*S*)sodium (0.2 mmol) and phenylbismuth dichloride (0.1 mmol) in absolute THF was heated under reflux with stirring for 24 h. Diethyl ether and hexane were added to this solution to precipitate the product, which was then recrystallized from a dichloromethane-hexane mixture (1:1 *v*/*v*). After 14 days large colorless block-shaped crystals of the title complex, suitable for X-ray diffraction analysis, were obtained.

### Refinement

All H atoms were placed geometrically and treated as riding on their parent atoms, with methylene C—H distances of 0.97Å and aromatic C—H distances of 0.93Å. The *U*_iso_(H) values were set at 1.2*U*_eq_(C).

### Crystal data

**Table:**

[Bi(C_6_H_5_)(C_5_H_10_NS_2_)Cl]	*F*(000) = 880
*M**_r_* = 469.79	*D*_x_ = 2.151 Mg m^−^^3^
Monoclinic, *P*2_1_/*c*	Mo *K*α radiation, λ = 0.71073 Å
*a* = 9.2029 (9) Å	Cell parameters from 3883 reflections
*b* = 18.1432 (17) Å	θ = 2.3–28.2°
*c* = 9.0779 (8) Å	µ = 12.60 mm^−^^1^
β = 106.811 (2)°	*T* = 298 K
*V* = 1451.0 (2) Å^3^	Block, colorless
*Z* = 4	0.23 × 0.22 × 0.21 mm

### Data collection

**Table:**

Bruker SMART CCD area-detector diffractometer	2444 independent reflections
Radiation source: fine-focus sealed tube	1877 reflections with *I* > 2σ(*I*)
graphite	*R*_int_ = 0.095
φ and ω scans	θ_max_ = 25.0°, θ_min_ = 2.6°
Absorption correction: multi-scan (SADABS; Sheldrick, 2003)	*h* = −10→10
*T*_min_ = 0.160, *T*_max_ = 0.177	*k* = −21→19
6443 measured reflections	*l* = −10→10

### Refinement

**Table:**

Refinement on *F*^2^	Primary atom site location: structure-invariant direct methods
Least-squares matrix: full	Secondary atom site location: difference Fourier map
*R*[*F*^2^ > 2σ(*F*^2^)] = 0.056	Hydrogen site location: inferred from neighbouring sites
*wR*(*F*^2^) = 0.149	H-atom parameters constrained
*S* = 0.92	*w* = 1/[σ^2^(*F*_o_^2^) + (0.0942*P*)^2^] where *P* = (*F*_o_^2^ + 2*F*_c_^2^)/3
2444 reflections	(Δ/σ)_max_ < 0.001
147 parameters	Δρ_max_ = 2.04 e Å^−^^3^
0 restraints	Δρ_min_ = −2.84 e Å^−^^3^

### Special details

**Table:**

Geometry. All e.s.d.'s (except the e.s.d. in the dihedral angle between two l.s. planes) are estimated using the full covariance matrix. The cell e.s.d.'s are taken into account individually in the estimation of e.s.d.'s in distances, angles and torsion angles; correlations between e.s.d.'s in cell parameters are only used when they are defined by crystal symmetry. An approximate (isotropic) treatment of cell e.s.d.'s is used for estimating e.s.d.'s involving l.s. planes.
Refinement. Refinement of *F*^2^ against ALL reflections. The weighted *R*-factor *wR* and goodness of fit *S* are based on *F*^2^, conventional *R*-factors *R* are based on *F*, with *F* set to zero for negative *F*^2^. The threshold expression of *F*^2^ > σ(*F*^2^) is used only for calculating *R*-factors(gt) *etc*. and is not relevant to the choice of reflections for refinement. *R*-factors based on *F*^2^ are statistically about twice as large as those based on *F*, and *R*- factors based on ALL data will be even larger.

### Fractional atomic coordinates and isotropic or equivalent isotropic displacement parameters (Å^2^)

**Table:**

	*x*	*y*	*z*	*U*_iso_*/*U*_eq_	
Bi1	0.83539 (5)	0.19587 (2)	0.77837 (4)	0.0353 (2)	
Cl1	0.8497 (4)	0.32758 (19)	0.6005 (3)	0.0448 (8)	
N1	0.7954 (11)	−0.0056 (5)	0.4907 (10)	0.036 (2)	
S1	0.8233 (4)	0.14036 (18)	0.5054 (3)	0.0435 (8)	
S2	0.8036 (4)	0.0484 (2)	0.7681 (3)	0.0462 (8)	
C1	0.8051 (13)	0.0546 (7)	0.5771 (12)	0.040 (3)	
C2	0.7991 (16)	0.0000 (7)	0.3290 (12)	0.051 (3)	
H2A	0.8309	−0.0470	0.2976	0.061*	
H2B	0.8737	0.0366	0.3225	0.061*	
C3	0.6464 (14)	0.0208 (8)	0.2196 (11)	0.053 (4)	
H3A	0.5721	−0.0154	0.2255	0.079*	
H3B	0.6540	0.0227	0.1164	0.079*	
H3C	0.6163	0.0682	0.2477	0.079*	
C4	0.7770 (14)	−0.0790 (7)	0.5486 (14)	0.045 (3)	
H4A	0.8283	−0.0807	0.6581	0.054*	
H4B	0.8252	−0.1148	0.4985	0.054*	
C5	0.6136 (17)	−0.0998 (9)	0.5216 (18)	0.069 (4)	
H5A	0.5645	−0.0637	0.5679	0.103*	
H5B	0.6078	−0.1472	0.5667	0.103*	
H5C	0.5641	−0.1020	0.4130	0.103*	
C6	1.0885 (14)	0.1853 (6)	0.8490 (12)	0.035 (3)	
C7	1.1620 (16)	0.1381 (8)	0.9745 (13)	0.052 (4)	
H7	1.1050	0.1124	1.0271	0.063*	
C8	1.3156 (18)	0.1310 (9)	1.0167 (15)	0.063 (4)	
H8	1.3624	0.1002	1.0985	0.076*	
C9	1.4023 (18)	0.1673 (9)	0.9431 (17)	0.064 (4)	
H9	1.5074	0.1626	0.9761	0.077*	
C10	1.3332 (13)	0.2117 (8)	0.8179 (18)	0.055 (4)	
H10	1.3919	0.2351	0.7639	0.066*	
C11	1.1787 (15)	0.2213 (7)	0.7736 (15)	0.048 (3)	
H11	1.1339	0.2524	0.6915	0.058*	

### Atomic displacement parameters (Å^2^)

**Table:**

	*U*^11^	*U*^22^	*U*^33^	*U*^12^	*U*^13^	*U*^23^
Bi1	0.0391 (4)	0.0378 (4)	0.0300 (3)	0.00102 (19)	0.0113 (2)	−0.00018 (16)
Cl1	0.059 (2)	0.0432 (18)	0.0374 (14)	0.0086 (16)	0.0221 (14)	0.0032 (13)
N1	0.035 (6)	0.034 (6)	0.039 (5)	0.008 (5)	0.009 (4)	0.003 (4)
S1	0.064 (2)	0.0374 (18)	0.0303 (13)	−0.0063 (16)	0.0152 (13)	0.0013 (12)
S2	0.061 (2)	0.043 (2)	0.0348 (14)	−0.0054 (17)	0.0142 (14)	0.0066 (13)
C1	0.040 (7)	0.043 (8)	0.037 (6)	0.002 (6)	0.010 (5)	0.005 (5)
C2	0.073 (10)	0.043 (8)	0.039 (6)	0.014 (7)	0.022 (6)	−0.003 (5)
C3	0.060 (10)	0.048 (9)	0.046 (7)	0.008 (7)	0.008 (6)	0.003 (5)
C4	0.039 (8)	0.030 (7)	0.063 (7)	−0.001 (6)	0.012 (6)	0.002 (6)
C5	0.073 (11)	0.052 (10)	0.093 (10)	−0.010 (9)	0.042 (9)	0.009 (8)
C6	0.041 (8)	0.032 (7)	0.037 (6)	0.009 (5)	0.019 (5)	−0.002 (5)
C7	0.058 (9)	0.057 (10)	0.043 (6)	0.021 (7)	0.016 (6)	0.005 (6)
C8	0.069 (11)	0.068 (11)	0.051 (7)	0.031 (9)	0.016 (7)	0.004 (7)
C9	0.046 (9)	0.067 (11)	0.071 (9)	0.006 (8)	0.000 (8)	−0.028 (9)
C10	0.012 (7)	0.059 (10)	0.094 (10)	−0.009 (6)	0.016 (7)	−0.006 (8)
C11	0.055 (9)	0.039 (8)	0.055 (7)	−0.012 (7)	0.023 (6)	0.000 (6)

### Geometric parameters (Å, °)

**Table:**

Bi1—C6	2.238 (13)	C4—C5	1.500 (17)
Bi1—S1	2.647 (3)	C4—H4A	0.9700
Bi1—S2	2.690 (4)	C4—H4B	0.9700
Bi1—Cl1	2.908 (3)	C5—H5A	0.9600
Bi1—Cl1^i^	2.920 (3)	C5—H5B	0.9600
Cl1—Bi1^ii^	2.920 (3)	C5—H5C	0.9600
N1—C1	1.332 (15)	C6—C11	1.383 (16)
N1—C4	1.459 (14)	C6—C7	1.429 (16)
N1—C2	1.481 (13)	C7—C8	1.360 (19)
S1—C1	1.714 (13)	C7—H7	0.9300
S2—C1	1.742 (10)	C8—C9	1.35 (2)
C2—C3	1.516 (15)	C8—H8	0.9300
C2—H2A	0.9700	C9—C10	1.39 (2)
C2—H2B	0.9700	C9—H9	0.9300
C3—H3A	0.9600	C10—C11	1.373 (16)
C3—H3B	0.9600	C10—H10	0.9300
C3—H3C	0.9600	C11—H11	0.9300
			
C6—Bi1—S1	89.7 (3)	N1—C4—C5	112.7 (11)
C6—Bi1—S2	91.1 (3)	N1—C4—H4A	109.0
S1—Bi1—S2	67.33 (9)	C5—C4—H4A	109.0
C6—Bi1—Cl1	91.1 (3)	N1—C4—H4B	109.0
S1—Bi1—Cl1	77.84 (9)	C5—C4—H4B	109.0
S2—Bi1—Cl1	145.09 (8)	H4A—C4—H4B	107.8
C6—Bi1—Cl1^i^	87.6 (3)	C4—C5—H5A	109.5
S1—Bi1—Cl1^i^	149.27 (10)	C4—C5—H5B	109.5
S2—Bi1—Cl1^i^	82.11 (8)	H5A—C5—H5B	109.5
Cl1—Bi1—Cl1^i^	132.80 (6)	C4—C5—H5C	109.5
Bi1—Cl1—Bi1^ii^	116.11 (11)	H5A—C5—H5C	109.5
C1—N1—C4	122.1 (9)	H5B—C5—H5C	109.5
C1—N1—C2	120.7 (10)	C11—C6—C7	117.6 (12)
C4—N1—C2	117.2 (9)	C11—C6—Bi1	122.8 (9)
C1—S1—Bi1	88.4 (4)	C7—C6—Bi1	119.5 (9)
C1—S2—Bi1	86.5 (4)	C8—C7—C6	119.7 (13)
N1—C1—S1	121.2 (8)	C8—C7—H7	120.2
N1—C1—S2	121.0 (9)	C6—C7—H7	120.2
S1—C1—S2	117.8 (7)	C7—C8—C9	122.0 (14)
N1—C2—C3	112.5 (10)	C7—C8—H8	119.0
N1—C2—H2A	109.1	C9—C8—H8	119.0
C3—C2—H2A	109.1	C8—C9—C10	119.4 (15)
N1—C2—H2B	109.1	C8—C9—H9	120.3
C3—C2—H2B	109.1	C10—C9—H9	120.3
H2A—C2—H2B	107.8	C11—C10—C9	120.2 (14)
C2—C3—H3A	109.5	C11—C10—H10	119.9
C2—C3—H3B	109.5	C9—C10—H10	119.9
H3A—C3—H3B	109.5	C6—C11—C10	121.0 (13)
C2—C3—H3C	109.5	C6—C11—H11	119.5
H3A—C3—H3C	109.5	C10—C11—H11	119.5
H3B—C3—H3C	109.5		
			
C6—Bi1—Cl1—Bi1^ii^	90.5 (3)	C1—N1—C2—C3	−81.2 (14)
S1—Bi1—Cl1—Bi1^ii^	1.09 (12)	C4—N1—C2—C3	97.5 (13)
S2—Bi1—Cl1—Bi1^ii^	−2.9 (2)	C1—N1—C4—C5	89.4 (14)
Cl1^i^—Bi1—Cl1—Bi1^ii^	178.29 (5)	C2—N1—C4—C5	−89.2 (13)
C6—Bi1—S1—C1	91.2 (5)	S1—Bi1—C6—C11	60.6 (10)
S2—Bi1—S1—C1	−0.1 (4)	S2—Bi1—C6—C11	127.9 (10)
Cl1—Bi1—S1—C1	−177.6 (4)	Cl1—Bi1—C6—C11	−17.2 (10)
Cl1^i^—Bi1—S1—C1	6.4 (5)	Cl1^i^—Bi1—C6—C11	−150.0 (10)
C6—Bi1—S2—C1	−89.1 (5)	S1—Bi1—C6—C7	−117.6 (9)
S1—Bi1—S2—C1	0.1 (4)	S2—Bi1—C6—C7	−50.3 (9)
Cl1—Bi1—S2—C1	4.3 (4)	Cl1—Bi1—C6—C7	164.6 (9)
Cl1^i^—Bi1—S2—C1	−176.6 (4)	Cl1^i^—Bi1—C6—C7	31.8 (9)
C4—N1—C1—S1	−178.9 (8)	C11—C6—C7—C8	1.0 (19)
C2—N1—C1—S1	−0.3 (15)	Bi1—C6—C7—C8	179.3 (10)
C4—N1—C1—S2	2.4 (15)	C6—C7—C8—C9	0(2)
C2—N1—C1—S2	−179.0 (8)	C7—C8—C9—C10	−2(2)
Bi1—S1—C1—N1	−178.6 (10)	C8—C9—C10—C11	3(2)
Bi1—S1—C1—S2	0.1 (7)	C7—C6—C11—C10	0.1 (19)
Bi1—S2—C1—N1	178.6 (10)	Bi1—C6—C11—C10	−178.2 (10)
Bi1—S2—C1—S1	−0.1 (6)	C9—C10—C11—C6	−2(2)

Symmetry codes: (i) *x*, −*y*+1/2, *z*+1/2; (ii) *x*, −*y*+1/2, *z*−1/2.
